# Ranavirus Amplification in Low-Diversity Amphibian Communities

**DOI:** 10.3389/fvets.2022.755426

**Published:** 2022-02-09

**Authors:** Joe-Felix Bienentreu, Danna M. Schock, Amy L. Greer, David Lesbarrères

**Affiliations:** ^1^Department of Biology, Laurentian University, Sudbury, ON, Canada; ^2^Sciences and Environmental Technology, Keyano College, Fort McMurray, AB, Canada; ^3^Department of Population Medicine, University of Guelph, Guelph, ON, Canada

**Keywords:** amphibian decline, host richness, host identity, boreal forest, Canada, ranavirus, amplification

## Abstract

In an era where emerging infectious diseases are a serious threat to biodiversity, epidemiological patterns need to be identified, particularly the complex mechanisms driving the dynamics of multi-host pathogens in natural communities. Many amphibian species have faced unprecedented population declines associated with diseases. Yet, specific processes shaping host-pathogen relationships within and among communities for amphibian pathogens such as ranaviruses (RV) remain poorly understood. To address this gap, we conducted a comprehensive study of RV in low-diversity amphibian communities in north-western Canada to assess the effects of biotic factors (species identity, species richness, abundance) and abiotic factors (conductivity, pH) on the pathogen prevalence and viral loads. Across 2 years and 18 sites, with communities of up to three hosts (wood frog, *Rana sylvatica*; boreal chorus frog, *Pseudacris maculata*; Canadian toad, *Anaxyrus hemiophrys*), we observed that RV prevalence nearly doubled with each additional species in a community, suggesting an amplification effect in aquatic, as well as terrestrial life-history stages. Infection intensity among infected wood frogs and boreal chorus frogs also significantly increased with an increase in species richness. Interestingly, we did not observe any effects of host abundance or abiotic factors, highlighting the importance of including host identity and species richness when investigating multi-host pathogens. Ultimately, only such a comprehensive approach can improve our understanding of complex and often highly context-dependent host-pathogen interactions.

## Introduction

Host-pathogen interactions in natural communities are complex and context-dependent (1, 2). Pathogens can influence community processes at different trophic levels, including predator-prey interactions (3), inter-species competition (4), and social behavior. This includes, but is not limited to mate choice (5), avoidance of infected conspecifics (6), and higher activity rates in infected individuals (7). The identity and richness of suitable host species in a community can also influence infection prevalence and transmission of a pathogen, but effects are rarely straightforward (2, 8). The mode of transmission (e.g., frequency- vs. density-dependent), and individual traits such as life history and health of the host can also influence host-pathogen dynamics (2, 9–12). Overall, much remains to be explained regarding the transmission dynamics, and specific host-pathogen interactions of infectious diseases in natural systems, and there is still limited understanding of how pathogens shape host communities, and vice-versa.

In general, pathogens can be classified as either generalist or specialist with respect to the number of host species they infect (13, 14). While specialist pathogens are often highly adapted to evade the immune system of a particular host, such specific adaptations can be detrimental for generalist multi-host pathogens due to functional trade-offs (15, 16). Yet, the majority of known pathogens infect more than one host species because of the advantages of facilitated invasion and transmission when infecting multiple host species in a community, rather than relying on a single route of transmission (15, 17). Pathogen transmission can be characterized as either density-dependent, where the relative contact rate between infected and susceptible individuals is a function of the population density, or frequency dependent, where the transmission rates are independent from the population density (18). If and how pathogen transmission is dependent on host density is fundamental to pathogen dynamics in natural systems (19) and determines whether a pathogen is capable of causing the decline of a host species (20–22). Additionally, the richness, diversity, and population dynamics of host species in an ecosystem can have a significant influence on pathogen presence, prevalence and transmission (2, 8, 23–25). When multi-host pathogens are involved, disease risk may escalate in low diversity ecosystems, but decrease in diverse ecosystems. This so called dilution effect was first investigated in vector-borne pathogen systems [e.g., *Borrelia burgdorferi*, (26), *West Nile virus*, (27)], but in recent years there has been an increasing number of studies in directly transmitted pathogen systems, in particular hantaviruses (8, 28–30). The opposite mechanism, called amplification effect, has also been observed whereby higher diversity amplifies the disease risk [e.g., *Laguna Negra virus*: (31); *Louping ill virus*: (32)]. Interestingly, it has been shown that dilution and amplification effects can actually occur simultaneously in the same system, where pathogen prevalence depends on the respective strength and interactions of the two effects (8). Thus, pathogen prevalence appears to be variously amplified or diminished by host richness, abundance, and the sequence in which host communities are assembled and disassembled (2, 25, 33, 34).

Pathogens are a natural part of ecosystems, but in the last few decades an increasing number of pathogen-induced mass die-offs and associated declines have been observed in vertebrate populations. On a global scale, amphibians have experienced the most severe disease-associated declines (35–37), most often linked to the chytrid fungi *Batrachochytrium dendrobatidis* (Bd) and *B*. *salamandrivorans* (38, 39), and viruses in the genus *Ranavirus* (40, 41). Infections with these pathogens can cause potentially lethal systemic diseases, including but not limited to metabolic dysfunctions and organ failure (42–45). Chytrid fungi and ranaviruses (RVs) are both capable of infecting multiple host species within a community (43, 46, 47), and their broad host ranges likely have significant influences on pathogen dynamics. Unfortunately, the effects of species identity and species richness are not commonly studied in wild amphibian host-pathogen systems [see (34, 48)]. For instance, RVs are globally distributed multi-host pathogens, infecting a variety of different host species across three classes of ectothermic vertebrates [amphibians, reptiles, and fish; (43, 49)] but studies investigating RV dynamics tend to focus on selected subsets or single amphibian hosts (and life-history stage), often within a community of several host species [reviewed in (50)]. Susceptibility and infection outcome vary greatly and typically depend on host identity as well as the life-history stage of the infected individual (11, 12). In amphibians, Ranids (true frogs) and Hylids (tree frogs) often show higher susceptibility, than other families, and aquatic life-history stages in particular (e.g., larvae, tadpoles, and metamorphs) often exhibit greater susceptibility to lethal infections when compared to post-metamorphic individuals (12, 51). To our knowledge, only two previous studies investigated community-level effects in an amphibian-RV system. First, a study on wild Californian amphibian communities showed an increase in host richness amplified RV prevalence (52). Second, Rosa et al. (53) reported spatially and temporally confined amplification effects of host community diversity on RV prevalence in Spain, but no general or repeated patterns over several years could be identified.

Here, we combined extensive sampling with habitat and community assessments to test whether host species identity or richness affect pathogen prevalence and viral loads. We focused on the multi-host pathogen RV in low-diversity amphibian communities in north-western Canada. The presence of only three amphibian species in the research area allowed us to conduct an assessment of a whole host-pathogen system under natural conditions. We also examined the influence of relative host abundance and wetland-specific pH and conductivity, which are factors that have been found to influence the dynamics of other amphibian host-pathogen systems (1, 54–56). Following previous studies (34, 48, 52, 57, 58), we expected to observe either a dilution or an amplification effect related to species identity or richness, with less influence from host abundance or environmental variables on epidemiological patterns in this system.

## Materials and Methods

### Field Surveys and Sample Collection

**Figure 1:**
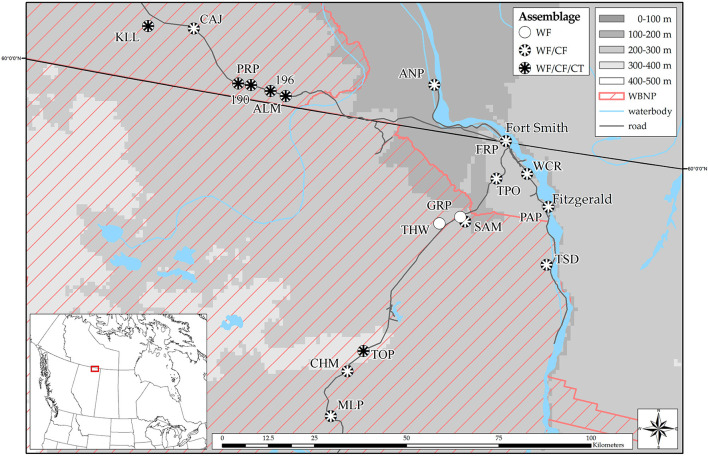
Amphibian communities sampled in Alberta and the Northwest Territories in 2016 and 2017 (WF, wood frog; CF, boreal chorus frog; CT, Canadian toad; WBNP, Wood Buffalo National Park). Map was created using ArcMap10.5 (Esri, Redlands, CA, USA).

**Figure 2:**
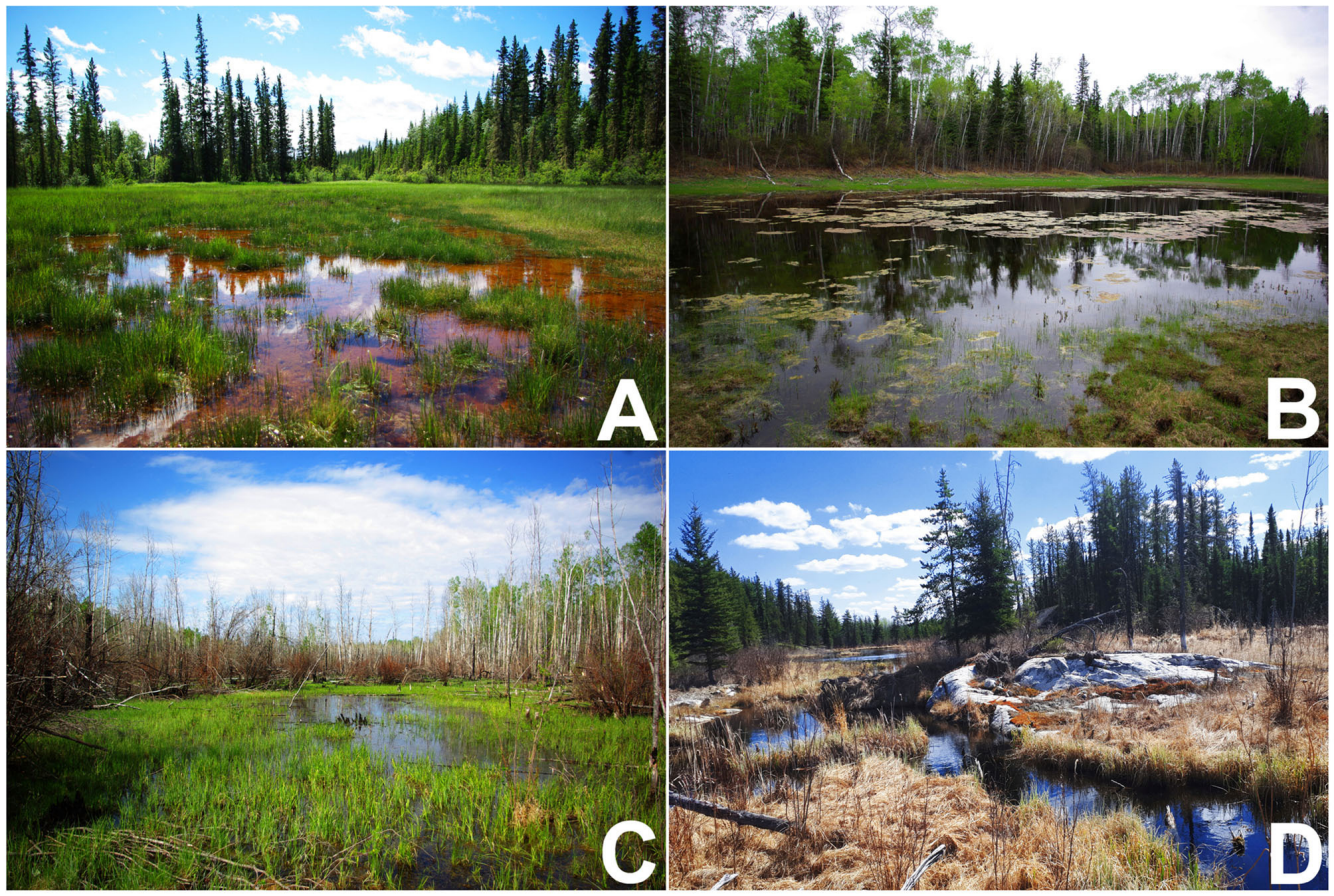
Representative amphibian habitats sampled in Alberta and the Northwest Territories in 2016 and 2017: Shallow marsh **(A)**, water-filled sinkhole **(B)**, floodplain **(C)**, and interconnected ponds and streams **(D)**. All photos by JF Bienentreu.

Field-work was conducted in north-eastern Alberta and the southern Northwest Territories, Canada, predominately within the boundaries of Wood Buffalo National Park (Figure 1). The area is part of the Taiga Plains and Boreal Plains Ecoregions, featuring a distinctive landscape consisting of extensive areas of interconnected river systems, wetlands and lakes, surrounded by boreal forest (59–61); (see Figures 2A–D). The area is characterized by a sub-humid climate with low mean annual air temperatures (62), a high rate of environmental disturbances (e.g., wildfires and floods), and ecoregion specific characteristics such as (semi-) permafrost (63, 64). Field reconnaissance surveys began the first week of May in 2016 and 2017 when spring melt occurs in the area, and continued until the end of June. This timing maximized our ability to acoustically detect all amphibian species expected in the area as well as physically encounter multiple life stages of each species. To determine the species present at each site and to estimate their abundance, we conducted a total of 221 visual, auditory, and dip netting surveys (75 in 2016; 146 in 2017) of suitable amphibian habitats such as floodplains, wetlands and meadows. Opportunistic surveys were conducted along the margins of the respective wetland, and down to an approximate water depth of 1 m. Three species of amphibians were encountered: wood frogs (*Rana sylvatica*, WF), boreal chorus frogs (*Pseudacris maculata*, CF), and Canadian toads (*Anaxyrus hemiophrys*, CT). A species was determined to be present at a site if the species was caught, or visually/auditorily identified even if we were unable to ultimately capture and collect tissue samples from the species at a site. Tadpoles of a given species were considered to be present when either visually confirmed, or when breeding behavior in adults was observed, and eggs were found. Throughout our extensive surveys we encountered three compositions: WF only, WF/CF, or all three species. Sites with CF or CT only, WF/CT, or CF/CT were never encountered.

A total of 18 unique sites were chosen for sampling (Figure 1; see Appendix I for full names and coordinates). Tissue sampling of terrestrial individuals (fully metamorphosed young of the year, juveniles, and adults) was conducted at 13 sites between May 15 and June 23, 2016. Tissue sampling of aquatic stages [tadpoles and metamorphs; Gosner stages 26 – 42 (65)] was conducted at 16 sites (11 identified in 2016 + 5 new in 2017) between June 14 and June 29, 2017. Due to logistical constraints, two of the sites sampled in 2016 were not sampled in 2017. To allow for comparisons of amphibian abundance among sites, we sampled each wetland using the same methodology, conducting opportunistic sampling for all species potentially present in the area. We quantified relative abundance as Catch Per Unit Effort (CPUE): the number of individuals encountered per unit of search time (here Ind./hour) according to (66). To allow for sufficient sample sizes for pathogen detection and statistical analysis, we selected sites with a minimum abundance of 10 terrestrials or 30 aquatic individuals per 1 h search time. If the required sample size could not be achieved at the first sampling, sites were sampled a second time within a 2 week time-frame of the initial sampling. To characterize abiotic characteristics of our study sites, temperature, water pH, and conductivity were recorded during each sampling event using an YSI 556 MPS (YSI Inc., Yellow Springs, United States), and were averaged if a site was sampled more than once. To more completely assess temperature profiles in our study wetlands, we deployed temperature data-loggers (Onset HOBO Fresh Water Data Logger U24-001) at three wetlands that differed in terms of size and other abiotic characteristics. The loggers were deployed 10 May−1 July, 2017, and were attached to a stationary pole approximately 50 cm below the water surface. Due to the landscape in the area, with vast areas of karst topography (i.e., sinkholes), it was not feasible to determine wetland depth.

Individual amphibians were caught by hand or by dip-netting and then held individually in new, single use plastic bags containing air and pond water (for aquatic stages), or wet vegetation (for terrestrial stages), until processed (<30 min). Care was taken to replenish air in the bags frequently and to protect the animals from direct sun and overheating. Aquatic individuals were measured (snout-vent-length; SVL) and staged (65) in their respective bag. Terrestrial individuals were weighed (g) and their SVL was measured. To test for RV infections, we non-lethally collected a ~1 mm toe-clip (terrestrials) or tail-clip (aquatic stages) from each individual. To prevent cross-contamination and spread of pathogens among individuals during handling, a new pair of nitrile gloves and a new scalpel blade was used for processing each animal, and all surfaces and tools were regularly disinfected with 99% isopropyl. Tissue samples were stored individually in 1.5 ml microcentrifuge tubes (filled with 95% ethanol, Eppendorf AG, Hamburg, Germany). To prevent accidental transfer of pathogens among sites, all gear and equipment that came into physical contact with the processed individuals, including nets, boots and waders were disinfected for 5 min with a 10% bleach solution, rinsed with clean tap water, and allowed to dry before being used at the next site [see (67)]. We solely used non-lethal tissue samples for ethical reasons and because they are easy to collect and store under remote field conditions. Tail and toe clips have been shown to be a viable alternative to lethal sampling and are in a generally high agreement with results from corresponding organ samples, but often slightly underestimate infection prevalence (44, 68–71). There are no known long-lasting negative effects on the amphibians when a single phalanx or tail clip is collected aseptically (68–70), and non-lethal sampling significantly decreases the impact on biodiversity, particularly when rare or threatened species are studied (44, 69, 72).

### PCR-Based Ranavirus Detection and Quantification

Ranavirus detection and quantification was performed using quantitative PCR (qPCR) following the cycling conditions described by Leung et al. (73), and quantification described by Hoverman et al. (11). DNA was extracted from toe and tail clips using Qiagen DNEasy® Blood and Tissue kits according to manufacturer specifications (QIAGEN Inc., Valencia, CA, USA). A Synergy H1 Hybrid Multi-Mode Reader was used to quantify concentration of genomic DNA in each sample (BioTek, Winooski, VT, USA). Briefly, the qPCR mixture contained 10 μl TaqMan Universal PCR Master Mix 2X (Thermofisher Scientific, Waltham, MA, USA), 1 μl forward primer MCPRV_F-5GTCCTTTAACACGGCATACCT3 (10 μM), 1 μl reverse primer MCPRV_R-5ATCGCTGGTGTTGCCTATC3 (10 μM), and 0.05 μl TaqMan probe MCP_NFQ-5TTATAGTAGCCTRTGCGCTTGGCC3 (100 μM). Subsequently, we added 250 ng of template DNA and DNA grade water to a final reaction volume of 20 μl. The qPCR was performed using an Mx3005P QPCR System (Agilent Technologies, Santa Clara, CA, USA). Cycling conditions were 50°C for 2 min, 95°C for 10 min, and 50 cycles of 95°C for 15 s, and 60 C for 30 s. Samples were run in duplicate in 96-well-plates. Each 96-well-plate included a no-template control (molecular grade water), and a serial dilution of a known quantity of cultured FV3 (1 x 10^6^-10^1^ copies/μl) to create a standard curve with precise fit (R^2^ >0.95). Individuals were considered positive if the target DNA was clearly amplified in both of the duplicates (i.e., surpassed the respective cycle threshold). If only one of the two wells amplified, a third reaction was conducted to either confirm or dismiss the previous positive result. Standard curves from each plate were used to calculate viral load following Yuan et al. (74) and reported as viral copies/250 ng of gDNA, as recommended by Gray et al. (69). Due to large standard deviations, viral loads are reported as log10 copies/250 ng gDNA (hereafter “logVL”). Primers used in our study target a consensus Major Capsid Protein (MCP) sequence of the major amphibian associated RV isolates: FV3: GenBank No AY548484, (75); TFV: AF389451, (76); CMTV: JQ231222, (77); EHNV, FJ433873, (78), and allow for a high analytical sensitivity when used in combination with a TaqMan probe (73). Pathogen identification and infection was confirmed through immunohistochemistry and *in-situ* hybridization on a subset of collected tissues (79).

### Statistical Analyses

Statistical analyses were conducted using R 3.3.3 (80) in RStudio Version 1.1.383 (81). Sample sizes allowed for reasonable prevalence estimates of RV at each site [95% confident that prevalence was < ~5% if no animals tested positive at a given site; (82, 83)]. We conducted analyses on the community level including all three species. Analyses at the individual and population level were conducted for WF only because single species sites were found only for this species. Aquatic and terrestrial life stages were analyzed separately. We used Pearson's Chi-squared tests to compare prevalence (percentage of positive individuals in a community) among the three different community compositions and used *post-hoc* pairwise comparisons to determine significance. We conducted one-way analyses-of-variance (ANOVAs) to test for differences in mean viral load among communities. We used Tukey's HSD test to detect differences among means for ANOVAs where the overall null hypothesis was rejected. To analyze RV prevalence among populations and communities, we performed beta regressions using the package “betareg” (84) for beta-distributed, dependent percentage data. To assess the influence of predictor variables on viral loads at the individual, population and community level, we performed generalized linear mixed model regressions fitted with a Poisson family and log link, using the package “lme4” (85). Each level of analysis included site-specific conductivity, pH, and species richness (WF, WF/CF, WF/CF/CT), as well as total relative community abundance (number of individuals caught per person-hour) as fixed effects. Due to the wide range of measurements for conductivity, it was scaled into seven categories (low to high conductivity). Site was treated as a random effect in all mixed model analyses. We used the dredge function in the R package “MuMIn” to create a sub-set of best-supported models of all possible sub-models from ranavirus presence and infection global models (86). The best supported models were compared separately using Akaike's Information Criterion [AIC; (87, 88)]. Due to the overall small sample sizes, we used AIC corrected for small sample sizes (AICC) in all our analyses (87, 89). Model comparison and averaging were performed using R packages ‘MuMIn’ (86) and ‘AICcmodavg’ (88), and can be found in the Supplementary Material (see Appendices II, III).

## Results

### Amphibian Community and Environmental Assessments

Three amphibian species were encountered in three types of community composition (Figure 1, Tables 1, 2), and we sampled a total of 1,244 individuals across all species and sites (Table 3). Wood frogs occurred at all sites where amphibians were found and were the only species we observed by itself (*n* = 2 sites; Table 4). The most commonly encountered community consisted of WF and CF (*n* = 10 sites; Table 4). If the sites featured large, shallow areas and pools, and provided nearby suitable hibernacula sites (e.g., sand dunes and pits), we often found CT as well (*n* = 6 sites). Abundance varied greatly among the three amphibian species: in terrestrial phases (2016), an average of 12 WF were caught per hour (range 3–37 individuals/hour) across 13 sites, whereas an average of 4 CF were caught per hour (range 1–9 individuals/hour) across nine sites (Table 1). For terrestrial stages, CT were caught at three sites at an extremely low average rate (1 individual/hour) and were observed at only four sites overall (Tables 1, 4). For aquatic stages (2017), an average of 40 WF were caught per hour across 16 sites (range 4–60 individuals/hour) whereas an average of 14 CF were caught per hour across 11 sites (range 2–41 individuals/hour; Tables 2, 4). Aquatic stages of Canadian toads were caught at two sites at an average rate of 9 individuals per hour (range 8–10 individuals/hour). We observed CT breeding activities at four additional sites, but could not find any aquatic stages during sampling. Although abundance varied among species, CF abundance did not vary significantly across the communities in which they were found (WF/CF and WF/CF/CT) in either terrestrial or aquatic life stages. Wood frog abundance however was lower in WF/CF/CT communities as compared to the two less complex communities in both terrestrial and aquatic stages. Across all our study sites, the pH ranged from 6.18 to 7.98 in 2016 and 6.90 to 8.71 in 2017 (Tables 1, 2). Conductivity ranged from 73 μS/cm to 8,290 μS/cm in 2016 and 70 μS/cm to 7,132 μS/cm across all sites (Tables 1, 2). Although we collected water temperature at the time of tissue sampling, it was not included in our analyses. Single-point measurements of temperature under field conditions are highly biased by sampling time and environmental conditions. This observation was further supported by the marked daily variability in our temperature data collected at three selected wetlands, with the means not differing from one another (see Appendix IV for data-logger recordings). Individual measures such as snout-vent length, weight, and Gosner stage for aquatic individuals, did not have any statistical significance in preliminary model comparisons and were excluded from the final analyses.

**Table 1:** Field sites in Alberta and the Northwest Territories where terrestrial amphibian life stages were sampled for ranavirus in 2016.

**Site**	**μS/cm**	**pH**	**sr**	**Species**	**Prevalence**	**log10 VL**	**hem**	**ind./hr**
GRP	1,256 ± 250	7.41 ± 0.8	1	WF	2/30 (7%)	2.58 ± 0.11	0/30 (0%)	14
THW	215 ± 0	6.20 ± 0	1	WF	3/30 (10%)	2.50 ± 0.23	0/30 (0%)	17
ANP	1,168 ± 0	6.55 ± 0	2	WF	5/30 (17%)	2.57 ± 0.07	2/30 (6.7%)	23
				CF*	n/a	n/a	n/a	0
CAJ	960 ± 315	7.18 ± 0.03	2	WF	4/30 (13%)	2.90 ± 0.32	0/30 (0%)	12
				CF	1/30 (3%)	2.57 ± 0	0/30 (0%)	5
FRP	332 ± 0	7.34 ± 0	2	WF	0/30 (0%)	-	0/30 (0%)	37
				CF	0/30 (0%)	-	0/30 (0%)	6
MLP	290 ± 54	6.68 ± 0.75	2	WF	0/17 (0%)	-	0/17 (0%)	5
				CF	1/4 (25%)	2.49 ± 0	0/4 (0%)	1
PAP	493 ± 11	7.10 ± 0.08	2	WF	5/30 (17%)	2.82 ± 0.33	0/30 (0%)	11
				CF	2/20 (10%)	2.57 ± 0.04	0/20 (0%)	4
SAM	8,290 ± 0	6.87 ± 0	2	WF	0/3 (0%)	-	0/3 (0%)	5
				CF*	n/a	n/a	n/a	0
WCR	381 ± 45	7.46 ± 0.12	2	WF	5/30 (17%)	2.68 ± 0.34	0/30 (0%)	8
				CF	1/4 (25%)	2.65 ± 0	0/4 (0%)	1
ALM	822 ± 7	6.79 ± 0.08	3	WF	5/30 (17%)	3.03 ± 0.24	0/30 (0%)	8
				CF	0/8 (0%)	-	0/8 (0%)	1
				CT	0/14 (0%)	-	0/14 (0%)	1
PRP	724 ± 88	7.98 ± 0.06	3	WF	13/30 (43%)	2.99 ± 0.33	0/30 (0%)	3
				CF	1/20 (5%)	2.79 ± 0	1/20 (5%)	4
				CT	0/7 (0%)	-	0/7 (0%)	1
TOP	73 ± 10	6.4 ± 0.1	3	WF	3/30 (10%)	3.37 ± 0.04	3/30 (10%)	5
				CF	2/30 (7%)	3.02 ± 0.17	0/30 (0%)	9
				CT*	n/a	n/a	n/a	0
190	1,270 ± 108	7.58 ± 0.34	3	WF	3/19 (16%)	3.08 ± 0.20	2/19 (10.5%)	3
				CF	0/16 (0%)	-	0/16 (0%)	3
				CT	0/1 (0%)	-	0/1 (0%)	1

*The data includes site-specific mean (+/− SD) conductivity (μS/cm), temperature (°C), and pH, species richness (sr), species-specific sample sizes, abundance, ranavirus prevalence (%), viral load (VL), and percentage of individuals with hemorrhages (hem) in wood frogs (WF), boreal chorus frogs (CF) and Canadian toads (CT). Abundance is presented as individuals caught per person-hour. Viral loads are presented as mean log10 viral copies/250 ng of gDNA ± SD. If a species was determined to be present at a site, but ultimately not captured and sampled, values are stated as n/a and species is marked with an asterisk*.

**Table 2:** Field sites in Alberta and the Northwest Territories where aquatic amphibian life stages were sampled for ranavirus in 2017.

**Site**	**μS/cm**	**pH**	**sr**	**Species**	**Prevalence**	**log10 VL**	**hem**	**ind./hr**
GRP	1382	8.71	1	WF	4/30 (13%)	2.67 ± 0.21	0/30 (0%)	60
ANP	822	7.35	2	WF	7/30 (23%)	3.44 ± 0.32	0/30 (0%)	90
				CF*	n/a	n/a	n/a	0
CAJ	819	7.36	2	WF	13/30 (43%)	2.57 ± 0.26	13/30 (43.3%)	30
				CF	9/30 (30%)	2.55 ± 0.15	8/30 (26.7%)	30
CHM	525	7.00	2	WF	7/30 (23%)	2.50 ± 0.19	5/30 (16.7%)	15
				CF*	n/a	n/a	n/a	0
FRP	447	6.90	2	WF	4/30 (13%)	2.45 ± 0.31	1/30 (3.3%)	60
				CF	2/8 (25%)	2.40 ± 0.08	0/8 (0%)	5
PAP	443	6.99	2	WF	2/30 (7%)	2.43 ± 0.22	9/30 (30%)	53e
				CF	3/30 (10%)	2.53 ± 0.21	9/30 (30%)	15
SAM	2,147	7.53	2	WF	7/30 (23%)	2.37 ± 0.10	4/30 (13.3%)	60
				CF*	n/a	n/a	n/a	0
TPO	429	7.89	2	WF	16/30 (53%)	2.55 ± 0.23	7/30 (23.3%)	60
				CF*	n/a	n/a	n/a	0
TSD	884	7.04	2	WF	7/30 (23%)	3.02 ± 0.51	9/30 (30%)	60
				CF	3/8 (38%)	2.95 ± 0.07	2/8 (25%)	8
WCR	327	7.53		WF	5/30 (17%)	3.34 ± 0.16	11/30 (36.7%)	20
				CF*	n/a	n/a	n/a	0
ALM	484	7.12	3	WF	5/30 (17%)	3.03 ± 0.24	0/30 (0%)	8
				CF	0/8 (0%)	-	0/8 (0%)	1
	e			CT*	0/14 (0%)	-	0/14 (0%)	0
KLL	7,132	7.70	3	WF	13/13 (100%)	4.69 ± 0.41	5/13 (38.5%)	4
				CF	30/30 (100%)	3.60 ± 0.48	10/30 (33.3%)	10
				CT	10/30 (33%)	3.32 ± 0.19	1/30 (3.3%)	10
PRP	1,072	8.46	3	WF	0/35 (0%)	-	2/35 (5.7%)	9
				CF	3/32 (9%)	2.56 ± 0.33	1/32 (3.1%)	8
				CT	0/31 (0%)	-	0/31 (0%)	8
TOP	70	7.36	3	WF	13/30 (43%)	3.94 ± 0.40	7/30 (23.3%)	15
				CF	4/5 (80%)	3.61 ± 0.63	2/5 (40%)	3
				CT*	n/a	n/a	n/a	0
190	1,212	7.90	3	WF	10/30 (33%)	3.93 ± 0.39	18/30 (60%)	60
				CF	2/3 (67%)	3.95 ± 0.37	3/3 (100%)	2
				CT*	n/a	n/a	n/a	0
196	244	7.97	3	WF	17/21 (81%)	4.92 ± 0.41	17/21 (81%)	21
				CF	39/41 (95%)	4.38 ± 0.62	22/41 (53.7%)	41
				CT*	n/a	n/a	n/a	0

*The data includes site-specific conductivity, temperature, and pH, species richness (sr), species-specific sample sizes, abundance, ranavirus prevalence, viral load (VL), and percentage of individuals with hemorrhages (hem) in wood frog (WF), boreal chorus frog (CF) and Canadian toad (CT). Each site was sampled in a single day in 2017. Abundance is presented as individuals caught per person-hour. Viral loads are presented as mean log10 viral copies/250 ng of gDNA ± SD. If a species was determined to be present at a site, but ultimately not captured and sampled, values are stated as n/a and species is marked with an asterisk*.

**Table 3:** Amphibian species sampled in 2016 and 2017, with respective life-stage, number of sampling sites, sampled individuals, ranavirus prevalence (% +/− SD), and viral loads.

**Year**	**Species**	**Stage**	**Sites**	***n***	**Prevalence**	**log10 VL**	**hem**
2016	WF	terrestrial	13	323	19 ± 11	2.83 ± 0.34	2
	CF	terrestrial	9	162	9 ± 6	2.71 ± 0.22	1
	CT	terrestrial	3	22	0	-	0
2017	WF	aquatic	16	459	37 ± 28	3.42 ± 1.00	31
	CF	aquatic	10	217	49 ± 32	3.73 ± 0.85	33
	CT	aquatic	2	61	16 ± 0	3.32 ± 0.19	2

*Wood frog (WF), boreal chorus frog (CF), Canadian toad (CT), viral load (VL), percentage of individuals with hemorrhages (hem). Viral loads are presented as mean log10 viral copies/250 ng of gDNA ± SD*.

**Table 4:** Amphibian communities sampled in Alberta and the Northwest Territories in 2016 and 2017, with respective life-stage, number of sampling sites, sampled individuals (n), ranavirus prevalence (% +/− SD) and viral load.

**Year**	**Composition**	**Stage**	**Sites**	***n***	**Prevalence**	**log10**
2016	**WF**	terrestrial	2	60	9 ± 2	2.53 ± 0.18
	**WF/CF**	terrestrial	7	258	15 ± 4	2.70 ± 0.27
	**WF**/CF	terrestrial	7	170	16 ± 2	2.73 ± 0.29
	WF/**CF**	terrestrial	5	88	10 ± 7	2.57 ± 0.06
	**WF/CF/CT**	terrestrial	4	189	23 ± 16	3.07 ± 0.27
	**WF**/CF/CT	terrestrial	4	93	28 ± 16	3.11 ± 0.29
	WF/**CF**/CT	terrestrial	4	74	7 ± 1	2.94 ± 0.18
	WF/CF/**CT**	terrestrial	3	22	0	-
2017	**WF**	aquatic	1	30	13 ± 0	2.67 ± 0.21
	**WF/CF**	aquatic	9	346	28 ± 14	2.68 ± 0.40
	**WF**/CF	aquatic	9	270	29 ± 20	2.70 ± 0.43
	WF/**CF**	aquatic	4	76	26 ± 10	2.60 ± 0.22
	**WF/CF/CT**	aquatic	6	361	55 ± 27	4.03 ± 0.80
	**WF**/CF/CT	aquatic	6	159	53 ± 31	4.24 ± 0.83
	WF/**CF**/CT	aquatic	6	141	65 ± 33	3.94 ± 0.76
	WF/CF/**CT**	aquatic	2	61	17 ± 0	3.32 ± 0.19

*Community level data are followed by a breakdown by species (if successfully sampled at respective site). Underlined species names indicate species considered in respective row: wood frog (WF), boreal chorus frog (CF), Canadian toad (CT). Viral loads (VL) are presented as mean log10 viral copies/250 ng of gDNA ± SD*.

### Ranavirus Distribution and Prevalence

Ranavirus was detected in individuals at all sites sampled (2016, *n* = 13 and 2017, *n* = 16; Tables 1, 2) and in all species (Table 3). The overall prevalence of RV across all species and sites was 16% in terrestrial individuals (2016) and 32% in aquatic stages (2017; Table 4). The mean infection prevalence in WF was 19% in terrestrial stages, and 37% in aquatic stages (Table 3). In CF, the mean infection prevalence was 9% in terrestrial stages, and 49% in aquatic stages (Table 3). We did not detect any infected terrestrial CT and the mean infection prevalence in CT aquatic stages was 16% (Table 3). It is notable that all RV-positive CT aquatic stages were detected at a single site during a RV die-off involving WF and CF [Tables 2–4; see Forzán et al. (79) for histological data on a subset of collected individuals]. Among all terrestrial individuals sampled, only 1% exhibited typical signs of ranavirosis such as hemorrhages at the toe-tips and ventral surfaces of the abdomen (44); see Table 1 for population level data). By contrast, 29% of all aquatic individuals sampled exhibited typical signs of RV infection such as hemorrhages in developing hind legs, at the base of the tail, and on the abdomen (44); (98 WF, 63 CF, 1 CT; see Table 2 for population level data), In addition to the aforementioned signs, aquatic individuals (*n* = 72) sampled during two die-off events in 2017 commonly exhibited edema. We found no effects of specific sampling dates on prevalence data for aquatic individuals (Z = 1.61, *p* = 0.11) or terrestrial individuals (Z = 0.03, *p* = 0.98), and we did not detect any temporal trends (Appendices V–VII).

### Viral Loads

The mean viral loads among positive terrestrial WF and CF individuals were low (logVL < 2.83), and RV was not detected in terrestrial CT (Tables 1, 3). Viral loads in aquatic stages varied greatly, with more than half of the individuals (61%) exhibiting higher mean viral loads in comparison to terrestrials (logVL < 3.65, Tables 2, 3). Wood frog and CF aquatic stages sampled during mass die-off events (*n* = 72) exceeded the commonly observed viral loads (38 individuals with logVL > 3.65 and 34 individuals with logVL > 4.65).

### Ecological Correlates of Ranavirus Prevalence and Viral Loads in Terrestrial Amphibian Life Stages (2016)

**Figure 3:**
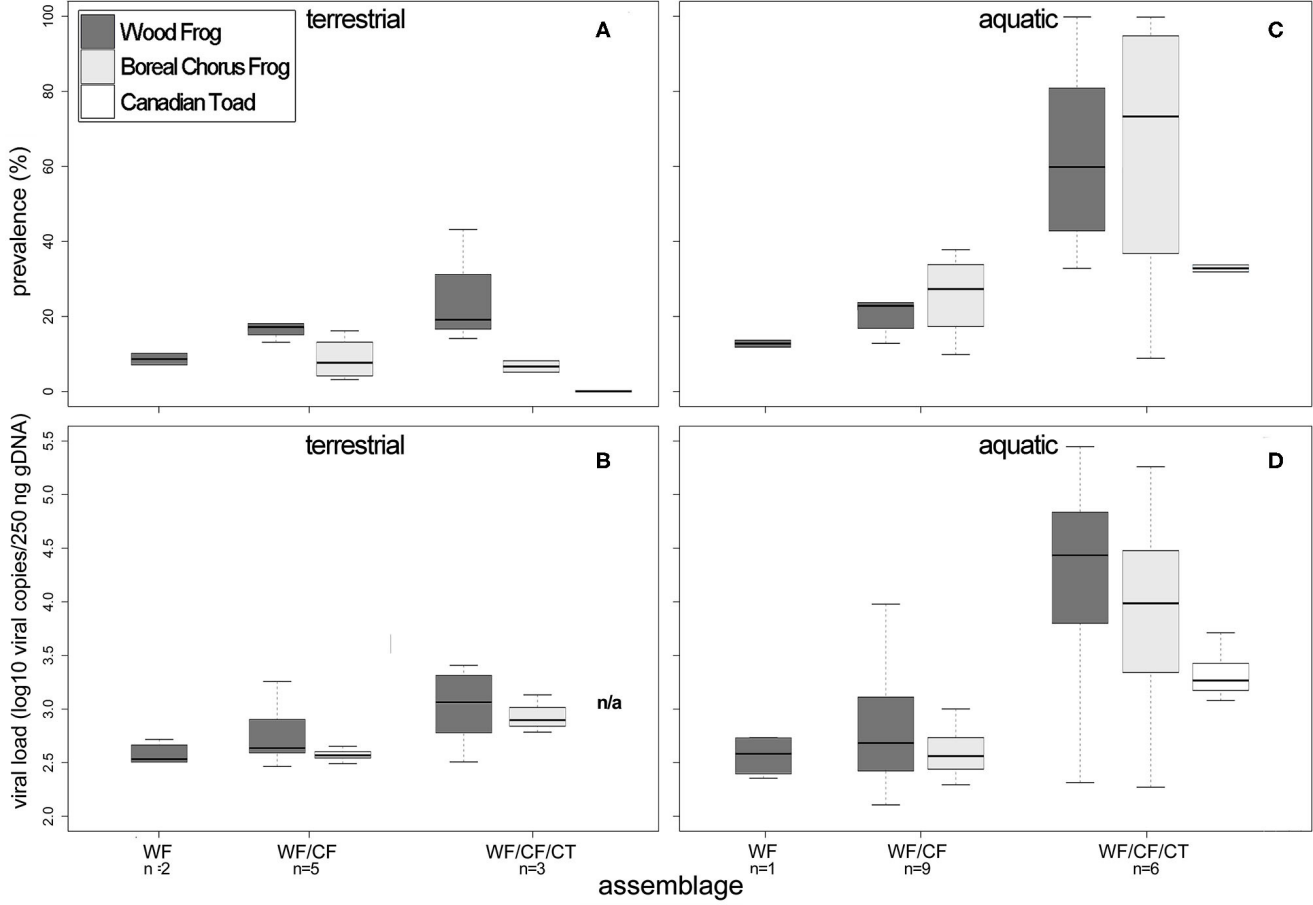
Min/max, median, and inter-quartile ranges of ranavirus infection prevalence (%) and viral load (log10 copies/250 ng of gDNA) in terrestrial **(A,B)** and aquatic stage **(C,D)** amphibians in northern Alberta and the Northwest Territories in 2016 and 2017, arranged by community assemblage. The number of sampled communities is stated below the respective assemblage. WF, Wood frog; CF, boreal chorus frog; CT, Canadian toad.

The percentage of RV positive individuals in a community increased with the number of species, but not significantly (χ^2^ = 2.19, df = 2, *p* = 0.335; Table 4, Figure 3A). At sites where WF occurred alone, we observed a 9% mean infection prevalence, increasing to 15% when CF were also present in the community. When three species were present, the mean community prevalence increased to 23% per site on average with regression analyses showing a significant effect of species richness in explaining the observed pattern, but no effects of other variables (Table 5, Appendix II). We also observed a significant increase in mean community viral loads linked to species richness (*F*_2, 7_ = 6.82, *p* < 0.05), while other variables had no explanatory power (Tables 4, 5, Figure 3B).

**Table 5:** Generalized linear mixed model (GLMR) and beta regression (BETA) results for ranavirus prevalence and viral loads in terrestrial amphibian life-stages at the community and population level (wood frog only).

	**Estimate**	**Std.Error**	**z value**	**Pr(>|z|)**	**Model type**
***IND_VL_WF***					GLMR
(Intercept)	5.150	0.535	9.29	<0.001	
Richness	0.745	0.158	4.55	<0.001	
Conductivity	−0.067	0.107	0.60	0.549	
pH	−0.085	0.321	0.26	0.797	
Abundance	−0.008	0.026	0.30	0.764	
***POP_PREV_WF***					BETA
(Intercept)	−1.590	0.795	2.00	<0.05	
Abundance	−0.019	0.033	0.60	0.549	
Conductivity	0.066	0.139	0.48	0.635	
pH	0.046	0.222	0.21	0.834	
richness	0.022	0.161	0.14	0.890	
***POP_VL_WF***					
(Intercept)	6.180	0.800	6.88	<0.001	GLMR
Richness	1.280	0.316	3.09	<0.01	
pH	0.502	0.387	1.04	0.299	
Abundance	−0.042	0.036	0.93	0.354	
Conductivity	0.027	0.192	0.11	0.911	
***COM _VL***					
(Intercept)	5.250	0.370	12.12	<0.001	GLMR
Richness	0.554	0.137	3.35	<0.001	
Abundance	0.002	0.025	0.06	0.956	
Conductivity	−0.019	0.108	0.14	0.887	
pH	0.093	0.273	0.29	0.769	
***COM _PREV***					BETA
(Intercept)	−2.360	0.583	4.04	<0.001	
Richness	0.148	0.212	0.70	<0.05	
pH	0.054	0.171	0.32	0.751	
Conductivity	0.012	0.072	0.16	0.872	
Abundance	−0.001	0.013	0.06	0.953	

*Individual (IND), population (POP), community (COM), viral load (VL), wood frog (WF). Please see Appendix II for detailed output from statistical models*.

At the population level (WF only), we observed a significant increase in infection prevalence from 9 (WF) to 16 (WF/CF), to 28% (WF/CF/CT; χ^2^ = 8.5, df = 2, *p* < 0.05; Table 4), but did not find any significant relationships of population prevalence and abiotic variables (Table 5). We also found a significant positive relationship of mean viral loads in terrestrial WF stages with the number of syntopic species in the community, at the individual and population levels (Table 5).

### Ecological Correlates of Ranavirus Prevalence and Viral Loads in Aquatic Amphibian Life Stages (2017)

In the aquatic amphibian life stages we observed that at the community level, ranavirus prevalence nearly doubled with each additional species (Table 4, Figure 3C). Wood frog aquatic stages in sites with no other amphibian species exhibited a low RV infection prevalence of 13% while in communities consisting of WF and CF we found significantly higher infection prevalence across the two species (28%; Table 4). When CT were also present in the communities, the mean community infection prevalence increased significantly to 55% across the three species (χ2 = 45.85, df = 2, *p* < 0.001; Table 4). Regression analyses showed significant effect of species richness on RV prevalence at the community level, and no effects of other variables (Table 6, Appendix III). We also observed a significant increase in mean community viral loads (*F*_2, 13_ = 1.963, *p* < 0.05) linked to species richness, while other variables had no explanatory power (Tables 4, 6, Figure 3D).

**Table 6:** Generalized linear mixed model (GLMR) and beta regression (BETA) results for ranavirus prevalence and viral loads in aquatic amphibian life stages at the community and population level (wood frogs only).

	**Estimate**	**Std.Error**	**z value**	**Pr(>|z|)**	**Model type**
***IND_VL_WF***					GLMR
(Intercept)	4.97	2.010	2.47	<0.05	
Richness	4.43	1.060	4.15	<0.001	
pH	0.317	0.453	0.70	0.486	
Abundance	0.001	0.004	0.19	0.853	
Conductivity	−0.011	0.067	0.16	0.872	
***POP_PREV_WF***					BETA
(Intercept)	−1.950	1.900	1.03	0.304	
Richness	2.120	1.360	1.55	<0.05	
Conductivity	0.061	0.126	0.48	0.630	
pH	0.160	0.387	0.41	0.680	
Abundance	−0.002	0.006	0.26	0.797	
***POP_VL_WF***					
(Intercept)	7.930	1.500	4.84	<0.001	GLMR
Richness	4.050	1.050	3.44	<0.001	
Abundance	0.001	0.005	0.22	0.825	
pH	0.070	0.275	0.24	0.813	
Conductivity	−0.018	0.072	0.23	0.819	
***COM _VL***					
(Intercept)	0.886	3.220	0.26	0.797	GLMR
Richness	2.680	0.947	2.63	<0.01	
pH	0.350	0.685	0.49	0.625	
Abundance	0.001	0.009	0.10	0.921	
Conductivity	−0.019	0.131	0.13	0.898	
***COM _PREV***					BETA
(Intercept)	−2.740	1.260	2.17	<0.05	
Richness	1.020	0.369	2.78	<0.01	
Abundance	0.005	0.0108	0.50	0.619	
Conductivity	−0.017	0.141	0.12	0.906	
pH	−0.061	0.367	0.17	0.868	

*Individual (IND), population (POP), community (COM), viral load (VL), wood frog (WF). Please see Appendix III for detailed output from statistical models*.

At the population level (WF only), we observed an increase in prevalence from 13% (WF) to 29% (WF/CF), and to 53% (WF/CF/CT; χ^2^ = 22.437, df = 2, *p* < 0.001). Regression analyses showed a significant positive correlation of population prevalence in WF aquatic stages with the number of syntopic species in the community, with no significant effect of other variables (Table 6). The same patterns were observed for viral loads in WF aquatic stages at individual and population levels (Table 6). When data collected during mass die-off events were excluded from the statistical analysis, species richness remained the main predictor for prevalence and viral loads on community level and WF population level (see Appendix VIII).

## Discussion

There is still limited knowledge of how pathogens are shaping communities and vice-versa, and much more remains to be explained regarding transmission dynamics, relationships, and specific interactions in multi host-pathogen systems. Our data are consistent with amplifying effects of community richness, whereby an increase in the number of host species was positively correlated with pathogen prevalence and viral loads. We also observed strong inter- and intraspecific differences prevalence and viral loads linked to host identity and life-history stage. However, we did not find evidence for any effect of abundance or any abiotic factors on epidemiological patterns, contrary to other amphibian pathogen systems [e.g., Bd (56, 90, 91)].

Epidemiological patterns in multi-host pathogen systems can be directly dependent on the specific competence and number of host species involved (14, 92, 93), but studies on such relationships are sparse in the amphibian disease literature. In general, studies often investigate single hosts, or focus on surveillance rather than investigating the underlying host-pathogen relationships. Two major amphibian pathogens (Bd and RV) infect multiple host species within a community (43, 46, 47). Therefore it is critical to include host identity and species richness as factors when investigating the pattern of these pathogens in the wild. Changes in host species composition and their respective competence can influence the dynamics (e.g., transmission and persistence) of pathogens present in the system but may also lead to the introduction of new pathogens into naïve host communities (12, 58, 94, 95). In our system, we observed increases of pathogen prevalence and viral loads in concert with an increase in species richness in both aquatic and terrestrial life stages. Therefore, it is possible that host identity and richness were key drivers of host-pathogen interactions in our system. Similarly, a large-scale study of amphibian communities in California found that an increase amphibian host richness significantly raised site-level RV prevalence (52), and a study on amphibian ranaviruses in southern Europe reported localized amplification effects but did not observe generalizable spatial or temporal relationships (53). Comparable relationships of specific host identity and pathogen dynamics were observed in other amphibian-host-pathogen systems [Bd, (57, 96); *Ribeiroia ondatrae*, (58)].

Habitat characteristics are regularly considered in epidemiological studies involving amphibian pathogens. In particular, temperature, pH, and conductivity can have effects on pathogen dynamics under specific circumstances [reviewed by Bienentreu and Lesbarrères (50)]. We included pH and conductivity as abiotic factors in our analysis but did not find any significant effects. Our findings are consistent with other amphibian-RV studies where abiotic factors did not appear to be directly driving RV dynamics (52, 91, 97). Nonetheless, water characteristics such as pH and conductivity can have effects on amphibian overall body condition, which in turn may indirectly influence pathogen dynamics (50, 98–100). It is important to consider that the effects of abiotic factors are often observed under controlled lab conditions, but field studies regularly fail to find specific relationships. Under natural conditions, pathogen dynamics may be affected by unknown or uninvestigated habitat characteristics. As well, animals move throughout their habitat in search of preferred microhabitat conditions. Temperature has been shown to influence ranavirus dynamics, with an increase in temperature causing faster viral replication rates (40, 51). However, temperature in wetlands fluctuates throughout a single day, including at the three wetlands where we placed temperature loggers in 2017 (Appendix IV). Our data show that it is not uncommon for temperatures to fluctuate more than 12°C at a single location in a single day. Given that amphibians explore their environment throughout the day in pursuit of food and thermal optima, and to evade predators, the effect of temperature on RV infections is difficult to assess accurately, particularly in small bodies of water (101).

While amplification effects in other host-pathogen systems have been directly linked to the number of (susceptible) hosts in a specified area (102), ranavirus epidemics in amphibians are generally not following this pattern. A field study of RV epizootics in wood frog populations in the eastern United States found no effects of host density on the pathogen dynamics (97), and research conducted on amphibian communities in California similarly found no density effects at the site level (52). These findings are further supported by experimental RV exposure trials, where an increase in density lowered mortality in wood frog tadpoles (103). Although many of our wetlands are either part of bigger, inter-connected wetland and river systems, or semi-permanent bodies of water with drastic fluctuations in water-levels making their size undetermined, we quantified relative abundance as Catch-Per-Unit Effort (66). Using this effective time count method to gather relative abundance data for frogs (104), we did not find any significant effects of host abundance on pathogen prevalence or viral loads in our RV-amphibian system. Even when data collected at the sites where die-offs took place [see Forzán et al. (79)] were excluded from the analyses, species richness remained the most parsimonious explanatory variable for viral loads at the individual, population, and community levels, regardless of the life-history stage. Therefore, it is plausible that die-off incidents in our study system are linked to the host identity and richness at a wetland rather than host density and/or abundance.

We found significant differences in inter- and intraspecific infection prevalence and viral loads, representing important indicators of the relative susceptibility of the host species (12). Among the three species present in our study system, WF and CF exhibited higher infection rates and viral loads than CT. Within species, post-metamorphic individuals exhibited considerably lower prevalence and viral loads than aquatic life-stages, and rarely showed typical signs of ranavirosis [e.g., lethargy, hemorrhages; see Miller et al. (44)]. Our findings are consistent with previous studies that demonstrated higher susceptibility to lethal RV infections in Ranids and Hylids than in Bufonids (51). Our findings are also consistent with other studies that demonstrated that larval amphibians are more likely to experience lethal RV infections than adults [(11, 12); see also (105) for multi-year data from our study region]. Notably, all positive CT tadpoles were sampled during a single die-off event of the other two species, but did not experience mass-mortality themselves [see (79)]. This could indicate spillover events from WF and/or CF into CT, only occurring when CT are exposed to a large number of infectious particles due to ongoing die-off events in other species.

Overall, our results also increase knowledge of RV distribution and dynamics in the Canadian north as the only other study in the region detected RV only in WF (94). A possible explanation for this discrepancy is the higher sensitivity of real-time qPCR methods used in our study relative to the conventional PCR methods used in the previous study (94). More than half of the positive individuals in our study exhibited low viral loads which could potentially lead to an increased number of false-negative samples with conventional PCR assays (70). The high percentage of sublethally infected individuals in our study area also supports the reservoir theory in amphibian-RV systems (51, 106), whereby low-susceptible host species or life stages can sustain sub-lethal infections and eventually infect individuals of other species in the long run. This possibility is supported by several experimental and field studies reporting sublethal RV infections (51, 103, 106–109) including an experimental study with WF collected in our study area wherein post-metamorphic individuals developed sub-lethal infections when exposed to environmentally relevant concentrations of RV virions via water bath (110). Wood frogs occur across our study region, and RV was found at all sampled wetlands and not restricted to a specific community of hosts. Therefore, it is possible that the WF in our system harbor low viral loads throughout hibernation and shed virions into the environment after returning to the wetlands in the spring, in turn infecting conspecifics and syntopic species in the community (106).

Due to their complexity, host-pathogen systems play a key role in many ecological communities. Our results underline the necessity for comprehensive habitat and community assessments in epidemiological studies involving multi-host pathogens. Ultimately, the combination of field studies with laboratory and/or mesocosm experiments will be critical to improve our understanding of the interactions between biodiversity and disease risk, providing further insights in managing wildlife pathogens that affect multiple host species.

## Data Availability Statement

The original contributions presented in the study are included in the article/Supplementary Material, and further inquiries can be directed to the corresponding author.

## Ethics Statement

This research was approved by Laurentian University Animal Care committee (protocol #2013-04-01). Wildlife research and collection permits were received from the Government of the Northwest Territories (Wildlife Research Permit WL500433 and WL500510), the Government of Alberta (Research Permits #57578 and #57829, Collection Licence #57579 and #57830) and Parks Canada (WB-2016-21358 and WB-2017-23740).

## Author Contributions

JFB, DS, and DL: conceptualization, methodology, and writing–original draft. JFB: data collection, curation, and visualization. JFB and AG: data analysis and validation. JFB and DL: funding acquisition. JFB, DS, AG, and DL: writing–review and editing. All authors contributed to the article and approved the submitted version.

## Funding

This research was funded by NSERC to DL (RGPIN-2018-06877) and the Ontario Trillium Scholarship to JFB.

## Conflict of Interest

The authors declare that the research was conducted in the absence of any commercial or financial relationships that could be construed as a potential conflict of interest.

## Publisher's Note

All claims expressed in this article are solely those of the authors and do not necessarily represent those of their affiliated organizations, or those of the publisher, the editors and the reviewers. Any product that may be evaluated in this article, or claim that may be made by its manufacturer, is not guaranteed or endorsed by the publisher.
