# Supplementary material for: Ranavirus Amplification in Low-Diversity Amphibian Communities
Source: Front Vet Sci. 2022 Feb 9;9:755426. doi: 10.3389/fvets.2022.755426 (PMC8863596; doi:10.3389/fvets.2022.755426)
Supplement: Supplementary file 1 [file Data_Sheet_1.PDF]

APPENDICES

Appendix I. Full names and geographic coordinates of amphibian sampling sites in northeastern Alberta and the Northwest Territories in 2016 and 2017. Species richness (sr).

Site	Name	N°	W°	sr	site description
GRP	Grosbeak Pond	59.801	-112.013	1	water-filled sinkhole
THW	Thunder Wetlands	59.778	-112.106	1	series of water-filled sinkholes of varying sizes
ANP	Antoinette's Pond	60.110	-112.251	2	small pond in old river bed
CAJ	Carmen's Journey	60.146	-113.453	2	small stream and flooded meadows
CHM	Cherry Mountains	59.383	-112.416	2	series of water-filled sinkholes of varying sizes
FRP	Frog Pond	60.002	-111.859	2	water-filled slough (shallow)
MLP	Mike's Lily Pond	59.267	-112.451	2	water-filled sinkhole
PAP	Paulette's Pond	59.859	-111.603	2	water-filled slough (shallow), flood-plain
SAM	Salt Marsh	59.798	-112.009	2	shallow marsh
TPO	T-Pond	59.907	-111.872	2	water-filled slough (shallow)
TSD	Tsá des (Beaver	59.717	-111.561	2	series of interconnected ponds and streams
WCR	Wolf Creek	59.930	-111.728	2	series of interconnected ponds and streams
ALM	Alfred's Marshlands	60.021	-112.949	3	vast fen-/marsh lands, series of small ponds
KLL	Klewi Lake	60.133	-113.683	3	large, shallow lake
PRP	Preble Pond	60.032	-113.189	3	water-filled slough (shallow)
TOP	Toadlet Pond	59.438	-112.356	3	water-filled sinkhole
190	Km190 HWY5	60.034	-113.126	3	series of interconnected ponds and streams
196	Km196 HWY5	60.028	-113.127	3	water-filled gravel pit (3 small ponds)

Appendix II. Generalized linear mixed model and beta regression model selection for ranavirus prevalence and viral loads in terrestrial amphibian life stages at the community and species level (wood frogs only). Model selections are based on AICc values. Individual (IND), population (POP), community (COM), viral load (VL), wood frog (WF).

Model selection based on AICc:	K	AICc	ΔAICc	AICcWt	Cum.Wt
IND_VL_WF					
Richness	4	7517.9	0	0.47	0.47
Richness_Conductivity	5	7520.4	2.49	0.14	0.61
Richness_pH	5	7520.5	2.58	0.13	0.74
Richness_Abundance	5	7520.6	2.71	0.12	0.86
Richness_Abundance_Conductivity	6	7522.9	4.99	0.04	0.9
POP_PREV_WF					
Abundance	3	-10.45	0	0.38	0.38
Conductivity	3	-10.12	0.32	0.33	0.71
pH	3	-9.38	1.07	0.22	0.93
Richness	4	-4.96	5.48	0.02	0.96
Abundance_Conductivity	4	-4.84	5.61	0.02	0.98
POP_VL_WF					
Richness	4	142.87	0	0.38	0.38
pH	3	143.68	0.81	0.26	0.64
Abundance	3	143.98	1.1	0.22	0.86
Conductivity	3	145.27	2.4	0.12	0.97
Abundance_PH	4	150.48	7.61	0.01	0.98
COM _VL					
Richness	3	141.97	0	0.84	0.84
Richness_abundance	4	147.82	5.85	0.04	0.88
Richness_Conductivity	4	147.89	5.93	0.04	0.92
Richness_pH	4	147.97	6	0.04	0.97
pH	3	150.11	8.15	0.01	0.98
COM _PREV					
Richness	3	-21.94	0	0.41	0.41
pH	3	-20.51	1.43	0.2	0.61
Conductivity	3	-20.01	1.93	0.16	0.76
Abundance	3	-19.87	2.07	0.14	0.91
Richness_Conductivity	4	-16.08	5.86	0.02	0.93

Appendix III. Generalized linear mixed model and beta regression model selection for ranavirus prevalence and viral loads in aquatic amphibian life stages at the community and species level (Wood frog only). Model selections are based on AICc values. Individual (IND), population (POP), community (COM), viral load (VL), wood frog (WF).

**Model selection based on AICc:**	**K**	**AICc**	**ΔAICc**	**AICcWt**	**Cum.Wt**
IND_VL_WF					
Richness	4	3E+06	0	0.3	0.3
Richness_pH	5	3E+06	0.49	0.23	0.53
Richness_Abundance	5	3E+06	1.79	0.12	0.65
Richness_Conductivity	5	3E+06	2.01	0.11	0.76
Richness_Abundance_PH	6	3E+06	2.52	0.08	0.85
POP_PREV_WF					
Richness	4	0.3	0	0.46	0.46
Richness_Conductivity	5	2.34	2.04	0.17	0.62
Richness_pH	5	2.96	2.66	0.12	0.74
Richness_Abundance	5	4.64	4.34	0.05	0.8
Abundance	3	5.03	4.73	0.04	0.84
POP_VL_WF					
Richness	4	306.93	0	0.66	0.66
Richness_Abundance	5	310.6	3.67	0.11	0.77
Richness_pH	5	310.61	3.69	0.11	0.87
Richness_Conductivity	5	310.67	3.74	0.1	0.98
Richness_Conductivity_PH	6	315.63	8.7	0.01	0.98
COM _VL					
Richness	3	333.81	0	0.49	0.49
Richness_pH	4	335.31	1.5	0.23	0.72
Richness_Abundance	4	337.14	3.34	0.09	0.81
Richness_Conductivity	4	337.26	3.45	0.09	0.9
Richness_Abundance_PH	5	339.41	5.61	0.03	0.93
COM _PREV					
Richness	3	-3.77	0	0.57	0.57
Richness_Abundance	4	-0.72	3.05	0.12	0.69
Richness_Conductivity	4	-0.16	3.62	0.09	0.78
Richness_pH	4	-0.16	3.62	0.09	0.88
Abundance	3	2.5	6.27	0.02	0.9

Appendix IV. Temperature logger data for three wetlands in Wood Buffalo National Park. Data were collected between May 10, 2017, and July 1, 2017. Black boxes indicate occasions when bears pulled the data loggers from the wetlands (those readings were excluded from mean and min.-max. temperature, and trend line calculations). Mean (+/- SD) temperatures in ^{o}C for the three ponds for which there were temperature logger data are as follows: GRP – 17.7 ± 3.7°C (10.0 – 29.5), TOP – 15.9 ± 4.0°C (6.4 – 27.1), and SAM – 16.9 ± 4.7°C (6.6 – 31.7).

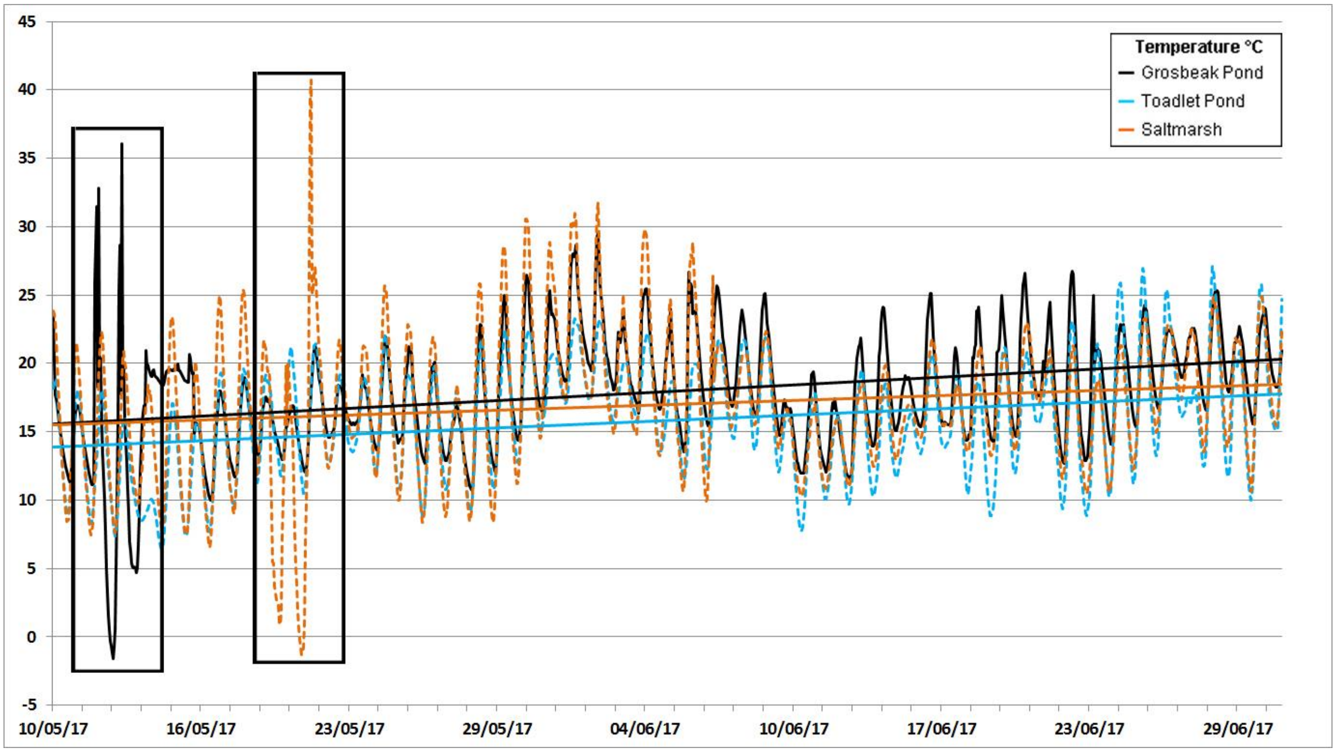

Appendix V. Ranavirus prevalence in relation to sampling date: (A) at the community level in terrestrial individuals; (B) at the species level in terrestrial individuals; (C) at the community level in aquatic individuals; (D) at the species level in aquatic individuals. Wood frog (WF), boreal chorus frog (CF), Canadian toad (CT), all three species (ALL).

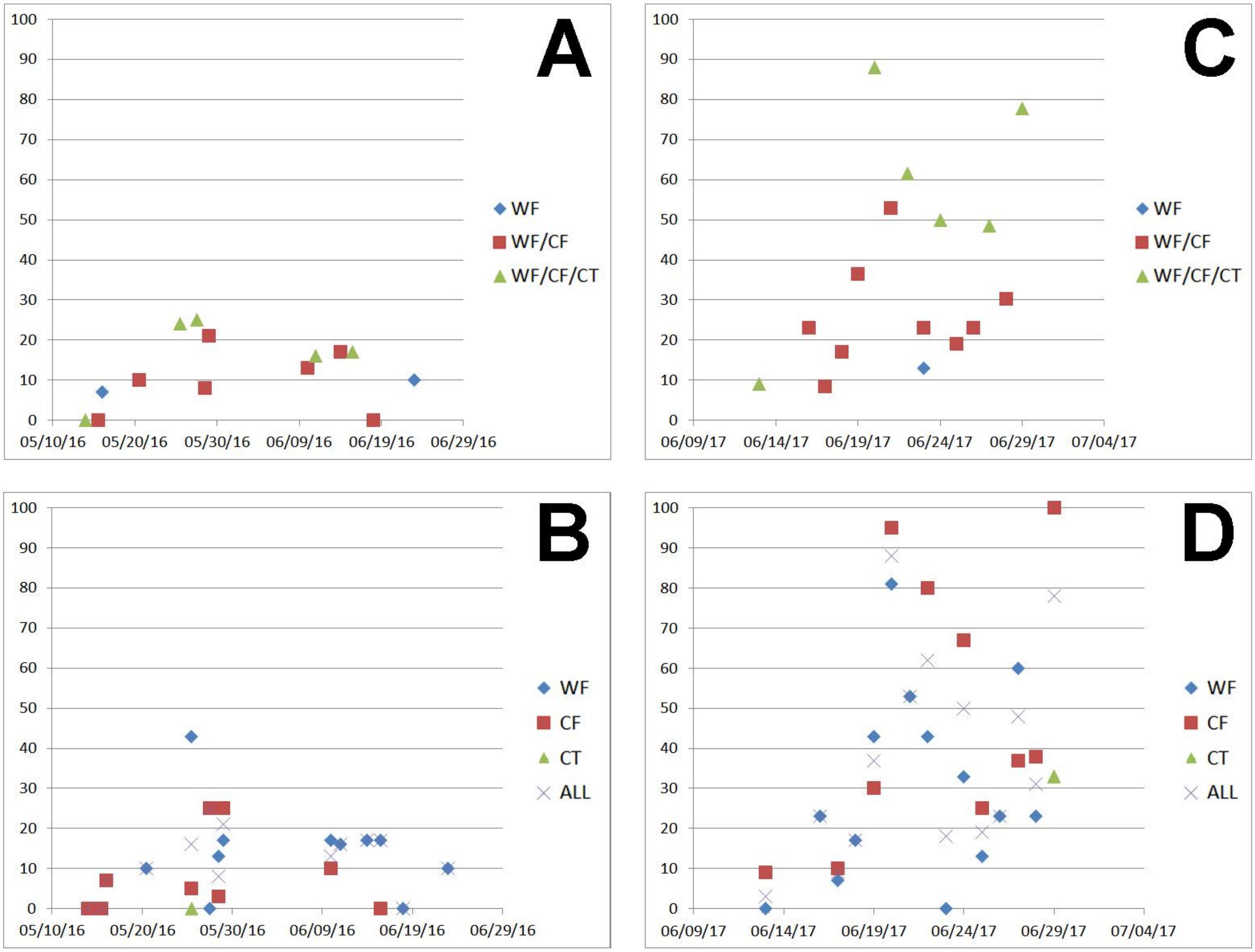

Appendix VI. Comparison of beta regression model selection for ranavirus prevalence in terrestrial and aquatic amphibian life history stages at the community level, with Julian date of tissue sampling included and Julian date of sampling date excluded. Model selections are based on AICc values. community (COM), prevalence (PREV), terrestrial (TER), aquatic (AQU), included (INCL).

**Model selection based on AICc:**	**K**	**AICc**	**ΔAICc**	**AICcWt**	**Cum.Wt**
COM _PREV_TER_NO DATE					
Richness	3	-21.94	0	0.41	0.41
pH	3	-20.51	1.43	0.2	0.61
Conductivity	3	-20.01	1.93	0.16	0.76
Abundance	3	-19.87	2.07	0.14	0.91
Richness_ Conductivity	4	-16.08	5.86	0.02	0.93
COM _PREV_TER_DATE INCL					
Richness	4	-22.18	0	0.34	0.34
Conductivity	4	-22.04	0.14	0.32	0.66
pH	4	-21.97	0.21	0.31	0.97
Richness_pH	5	-15.42	6.76	0.01	0.98
Richness_ Conductivity	5	-14.87	7.31	0.01	0.99
COM _PREV_AQU_NO DATE					
Richness	3	-3.77	0	0.57	0.57
Richness_Abundance	4	-0.72	3.05	0.12	0.69
Richness_ Conductivity	4	-0.16	3.62	0.09	0.78
Richness_pH	4	-0.16	3.62	0.09	0.88
Abundance	3	2.5	6.27	0.02	0.9
COM _PREV_AQU_DATE INCL					
Richness	5	-2.47	0	0.61	0.61
Richness_ Conductivity	6	-0.35	2.12	0.21	0.83
Richness_pH	6	2.14	4.61	0.06	0.89
Richness_Abundance	6	2.61	5.08	0.05	0.94
Abundance	4	4.71	7.18	0.02	0.95

Appendix VII Beta regression results of interactions between prevalence and Julian date in terrestrial and aquatic amphibian life history stages at the community level

COMMUNITY _PREVALENCE _TERRESTRIAL

Standardized weighted residuals 2:

Min	1Q	Median	3Q	Max
-1.7037	-0.7950	-0.1314	0.8581	1.7082

Coefficients (mean model with logit link):

	Estimate	Std. Error	z value	Pr(>|z|)
(Intercept)	4.698e+00	2.248e+02	0.021	0.983
Julian date	-4.174e-04	1.391e-02	-0.030	0.976

Type of estimator: ML (maximum likelihood)

Log-likelihood:	18.26 on 3 Df
Pseudo R-squared:	9.019e-05
Number of iterations:	29 (BFGS) + 7 (Fisher scoring)

COMMUNITY _PREVALENCE _AQUATICS

Standardized weighted residuals 2:

Min	1Q	Median	3Q	Max
-1.2816	-0.8629	-0.0831	0.4826	2.5088

Coefficients (mean model with logit link):

	Estimate	Std. Error	z value	Pr(>|z|)
(Intercept)	-1.406e+03	8.751e+02	-1.607	0.108
Julian date	8.184e-02	5.096e-02	1.606	0.108

Type of estimator: ML (maximum likelihood)

Log-likelihood:	3.915 on 3 Df
Pseudo R-squared:	0.1753
Number of iterations:	449 (BFGS) + 11 (Fisher scoring)

Appendix VIII. Generalized linear mixed model and beta regression results for ranavirus prevalence and viral loads in aquatic amphibian life stages at the community and species level (wood frogs only). Data collected during mass die-off events were excluded. Significance codes: *** 0.001, ** 0.01, * 0.05. Individual (IND), population (POP), community (COM), viral load (VL), wood frog (WF).

	Estimate	Std.Error	z_value	Pr(>|z|)
POP_PREV_WF				
(Intercept)	-1.32E+00	1.04E+00	1.26	2.07E-01
Richness	1.67E+00	7.89E-01	2.12	3.43E-02*
Abundance	-9.29E-03	8.74E-03	1.06	2.87E-01
pH	2.14E-01	3.57E-01	0.60	5.48E-01
Conductivity	-2.51E-02	1.26E-01	0.20	8.42E-01
POP_VL_WF				
(Intercept)	6.60E+00	1.16E+00	5.00	6.0E-07***
Richness	2.99E+00	1.03E+00	2.53	1.15E-02*
Conductivity	-1.44E-02	6.41E-02	0.21	8.37E-01
pH	3.49E-02	1.95E-01	0.16	8.71E-01
Abundance	3.65E-04	3.42E-03	0.10	9.25E-01
COM _VL				
(Intercept)	5.71E+00	1.17E+00	4.35	1.34E-05***
Richness	3.54E+00	9.77E-01	3.20	1.39E-03**
Conductivity	-2.71E-02	8.38E-02	0.30	7.62E-01
pH	3.15E-02	1.85E-01	0.15	8.78E-01
Abundance	4.84E-04	3.71E-03	0.12	9.06E-01
COM _PREV				
(Intercept)	-1.431636	8.69E-01	1.65	9.93E-02.
Richness	1.62E+00	7.00E-01	2.32	2.05E-02*
Abundance	-4.49E-03	8.76E-03	0.51	6.08E-01
Conductivity	-4.74E-02	1.12E-01	0.42	6.73E-01
pH	8.28E-03	3.16E-01	0.03	9.79E-01
